# Nucleus Accumbens Hyperactivity and mPFC–NAc Circuit Dysfunction Promote Self-Injurious Behavior in Rats

**DOI:** 10.3390/ijms27146256

**Published:** 2026-07-14

**Authors:** Yanmei Chen, Zhonghui Zuo, Di Luo, Yiling Ni, Liqiang Yang, Shicong Zhu, Jichuan Zhang

**Affiliations:** 1 Department of Basic Medicine, Medical School, Kunming University of Science and Technology, Kunming 650550, China; zuozhonghui0513@163.com (Z.Z.); di.luo@siat.ac.cn (D.L.); yiling_ni@fudan.edu.cn (Y.N.); okgood101@163.com (L.Y.); az870466678@gmail.com (S.Z.); 2 Shenzhen Key Laboratory of Precision Diagnosis and Treatment of Depression, Shenzhen Institute of Advanced Technology, Chinese Academy of Sciences, Shenzhen 518055, China

**Keywords:** self-injurious behavior, endopeduncular nucleus, nucleus accumbens, dopamine receptors, GABA receptors

## Abstract

Self-injurious behavior (SIB) is a devastating and potentially life-threatening action with high prevalence in adolescents and patients with neuropsychiatric disorders. Accumulating evidence indicates that disruptions in multiple cellular and circuit mechanisms underlie vulnerability to SIB. We used an inducible SIB rat model to study synaptic modification during SIB. At 0.5 and 1 h after bilateral injection of muscimol (1.0 μg/side) into the rat endopeduncular nucleus (EP, a rodent homolog of the internal globus pallidus (GPi)), which induced SIB in rats, expression of the α-amino-3-hydroxy-5-methyl-4-isoxazole-propionic acid receptor (AMPAR) subunit 1 (GluA1) and the phosphorylation of GluA1 at Ser831 and Ser845 were tested in the lateral habenula (LHb), ventral tegmental area (VTA), nucleus accumbens (NAc), amygdala, and medial prefrontal cortex (mPFC) of the rat brain. We also tested if modulation of NAc activity with a GABA_A_ receptor agonist or antagonist or dopamine receptor agonist or antagonist or inhibiting the mPFC–NAc pathway affected SIB in rats. At 1 h after EP inhibition, total GluA1 expression and phosphorylated GluA1 were decreased in the mPFC, VTA, and NAc, but were increased in the amygdala compared with control rats. When the EP was inhibited by 0.2 μg/side muscimol, hyperactivation of the NAc increased SIB in rats. However, if the EP was inhibited by 1.0 μg/side muscimol, hyperactivation of the NAc had no effects on SIB. Inhibiting the mPFC–NAc pathway increased wound areas in rats with SIB. At the onset of SIB, excitatory synaptic transmission is simultaneously dampened in the reward and control circuitry (VTA, NAc, mPFC) and potentiated in the aversion circuitry (amygdala), indicating that SIB is associated with molecular signatures suggestive of a shift from reward to threat processing. Hyperactivation of the NAc increased SIB incidence in rats, but administration of dopamine receptor agonists and antagonists into the NAc did not significantly affect the incidence of SIB in this study. These findings provide a novel mechanistic perspective on SIB, offering a basis for the treatment of SIB.

## 1. Introduction

Self-injurious behavior (SIB) is the abnormal behavior of harming one’s own body. The prevalence of SIB is about 4% in the general American population [1] and is much higher in adolescents and young adults [2]. The incidence of SIB in patients with mental disorders is up to 50% [3]. Severe SIB can be devastating and potentially life-threatening. SIB refers to any act of self-injury, including suicidal self-injury, developmental self-biting, and cultural ritual scarifications. Non-suicidal self-injury (NSSI), which is self-harm not explicitly intended to cause death, has become a widespread and highly harmful, yet poorly understood, phenomenon among adolescents [4]. NSSI serves a psychological regulation function, and was found to lie at about 4% in Germany [5]. Across 17 countries in North America, Australia, Europe, and Asia, the pooled lifetime prevalence of NSSI among 10–19-year-olds is 17.7% (21.4% for girls, 13.7% for boys), indicating NSSI is a global and broad public health issue [6]. Why people or animals perform SIB is largely unknown. The motivational triggers and precise neural mechanisms underlying SIB still require extensive research.

Psychological hypotheses suggest that dysregulated emotional experience contributes to the initiation and maintenance of NSSI. Subjects reporting NSSI indicated more reactivity, intensity, and perseveration of dispositional negative emotion [7]. Self-injury serves to alleviate negative emotions and decrease dissociation, emotional distress, and post-traumatic symptoms [1]. Biological studies have indicated that genetic variations, stress, substance addiction, or brain injury may all contribute to SIB [8]. SIB in several well-known genetic intellectual disability syndromes is noticeably higher than in individuals with normal IQ [9]. However, research into its neural mechanisms remains in its early stages, and the specific mechanisms are not yet understood [8]. Furthermore, most human studies on NSSI can only be conducted after the act has occurred, limiting research into the brain mechanisms involved during the act of self-harm. The lack of suitable animal models constitutes a major constraint in SIB research [10].

In the lab, SIB can also be induced in rodents by repeated administration of the monoamine agonist pemoline [11], by acute high-dose administration of methamphetamine or the L-type calcium channel agonist BayK 8644 [12], and by neonatal dopaminergic lesions induced by 6-hydroxydopamine [13]. However, each of these has distinct limitations. Our previous study showed that bilateral infusion of muscimol, a γ-aminobutyric acid (GABA) receptor agonist, into the rat endopeduncular nucleus (EP, a rodent homolog of the internal globus pallidus (GPi)) induces robust SIB in rats. Approximately 0.5–1 h after EP inhibition, rats exhibit behaviors such as biting their own paws, tails, and abdomen, which can persist for about 6 h [14]. The EP is one part of the basal ganglion that connects cortical and thalamic neural networks through a direct (GPi) and indirect (GPe) pathway [15]. It is mainly recognized as a brain area critical for motor function, and is a therapeutic target in Parkinson’s disease (PD) [16,17] and Huntington’s disease [18,19] to improve movement. However, why inhibition of the EP leads to SIB is unclear.

Recently, more non-motor functions of the EP were found [20], which might be related to the SIB induced by its inhibition. The EP receives and sends connections to various brain areas, such as the amygdala [21,22], hippocampus [23], and lateral habenula (LHb) [24]. The EP reciprocally connects with the amygdala [21,24,25,26]. Moderate NSSI shows elevated right amygdala activation to threat, and higher right amygdala activation can predict NSSI severity [4]. Besides the amygdala, the EP sends projections to the LHb, which is widely recognized as a key brain region to encode and regulate “aversion,” as LHb neurons are strongly activated in response to punishment and unexpected aversive events [27]. LHb is an important circuitry hub that regulates monoamine nuclei such as the ventral tegmental area (VTA), which is a primary brain region of the reward system. Psychiatric conditions marked by impairments in cognitive control often emerge during adolescence, when the prefrontal cortex (PFC) and its inputs undergo structural and functional maturation and are vulnerable to disturbance by external events [28]. It was found that cortex lesions can increase the severity of SIB in rats [29], and in severe NSSI adolescents, the resting-state functional connectivity between the amygdala and mPFC is lower, which suggests a possible role of the medial prefrontal cortex (mPFC) in SIB. All these studies implicate brain circuits governing emotion, reward, and cognitive control in the pathophysiology of SIB. Understanding the relationship between the EP and brain areas related to reward, emotional, and cognitive processing is important for further clarifying the function of the EP and why its inactivation results in SIB in rats.

GluA1 is a key subunit of the AMPA-type glutamate receptor in the postsynaptic membrane. Its expression levels and phosphorylation status directly determine the membrane transport efficiency, channel conductance, and synaptic localization of AMPA receptors. Ser831 phosphorylation enhances AMPA receptor channel conductance, directly amplifying postsynaptic membrane responsiveness to glutamate. Ser845 phosphorylation promotes AMPA receptor transport to and stable localization on the postsynaptic membrane, increasing receptor density at the synapse [30]. GluA1 and its phosphorylation detection serve as a good method for directly quantifying excitatory synaptic function. While other receptors such as NMDA receptor also reflect excitatory synaptic strength regulation, they are largely indirect indicators [31]. Enhanced excitatory synaptic function typically correlates with learning and memory formation/consolidation processes [32]. Alterations in excitatory synaptic function within brain regions such as the PFC and NAc directly regulate emotional and behavioral responses. For instance, enhanced excitatory synaptic function in the NAc correlates with reward-seeking behaviors and addiction, while diminished synaptic function in the PFC may impair emotional regulation, closely linking to normal emotional fluctuations such as post-stress mood changes [33,34].

In this study, we first characterized acute synaptic modifications during SIB onset by quantifying total GluA1 expression and phosphorylation states (Ser831/845) in the LHb, VTA, NAc, mPFC, and amygdala at 0.5 and 1 h following bilateral EP muscimol injection. Building upon our prior identification of the LHb–VTA circuit in SIB vulnerability [14], we subsequently tested whether the NAc functions as a downstream regulator by examining if bidirectional modulation of NAc neuronal activity via GABAergic or dopaminergic manipulations could alter the SIB. Finally, we investigated whether the mPFC–NAc pathway plays a role in SIB.

## 2. Results

### 2.1. EP Inactivation Changed the Excitatory Synaptic Input in Fronto-Limbic Brain Areas

**Figure 1:**
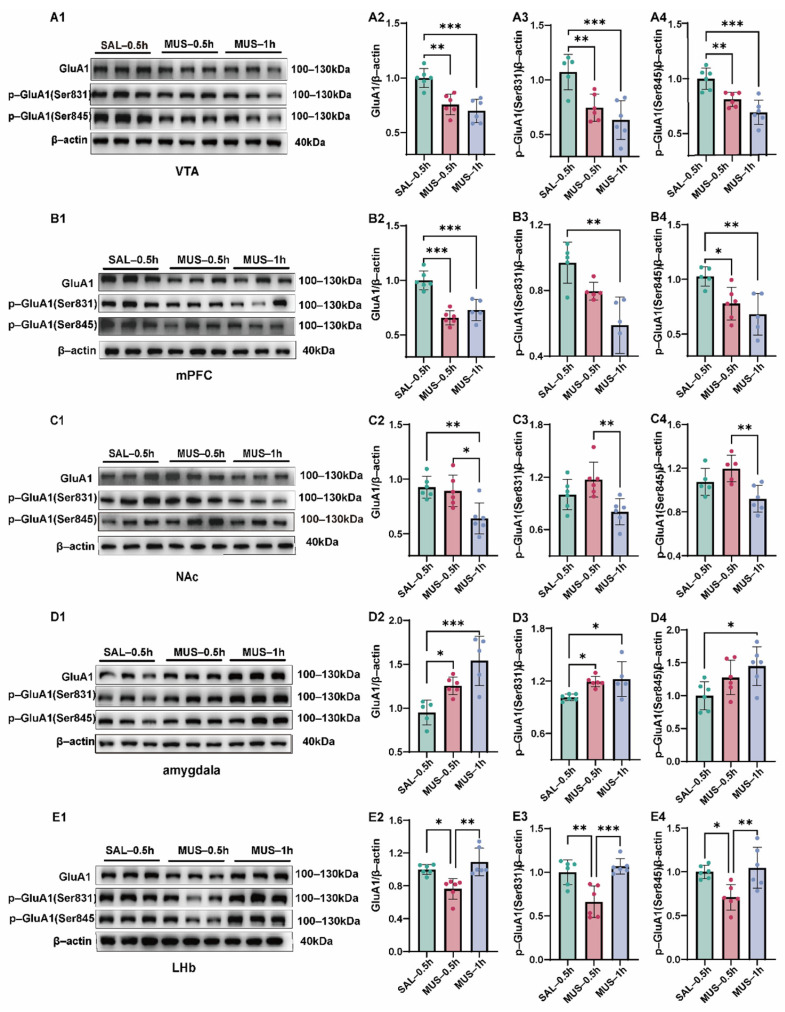
Synaptic modifications in VTA, mPFC, NAc, amygdala and LHb at the onset of SIB. The expression of GluA1 and GluA1 phosphorylation in VTA (**A1**–**A4**), in mPFC (**B1**–**B4**), in NAc (**C1**–**C4**), in amygdala (**D1**–**D4**), and in LHb are shown (**E1**–**E4**). Data are expressed as the mean ± SEM, *n* = 6 rats for each group. * *p* < 0.05, ** *p* < 0.01, *** *p* < 0.001 for difference between the control and drug-treated group. VTA: ventral tegmental area; PFC: prefrontal cortex; NAc: nucleus accumbens; LHb: lateral habenula; SIB: self-injurious behavior; GluA1: glutamate receptor AMPA-type subunit 1.

The expression of GluA1 significantly decreased in VTA and mPFC 0.5 h and 1 h after EP inactivation compared with the saline group (VTA: F (2,15) = 16.52, *p* < 0.001, Tukey: SAL–0.5 h vs. MUS–0.5 h, *p* = 0.001; SAL–0.5 h vs. MUS–1 h, *p* < 0.001, Figure 1(A1,A2); mPFC: F (2,14) = 28.44, *p* < 0.001, Tukey: SAL–0.5 h vs. 0.5 h, *p* = 0.001; SAL–0.5 h vs. 1 h, *p* < 0.001, Figure 1(B1,B2)). The phosphorylation of GluA1 at p831 and p845 in VTA and mPFC was also decreased by EP inactivation 0.5 h and 1 h later (p831: VTA: F (2,14) = 11.49, *p* = 0.001, Tukey: SAL–0.5 h vs. MUS–0.5 h, *p* = 0.01, Figure 1(A3); SAL–0.5 h vs. MUS–1 h, *p* < 0.001, Figure 1(A3); mPFC: F (2,12) = 11.31, *p* = 0.002, Tukey: SAL–0.5 h vs. MUS–1 h, *p* = 0.001, Figure 1(B3); p845: VTA: F (2,15) = 16.34, *p* < 0.001, Tukey: SAL–0.5 h vs. MUS–0.5 h, *p* = 0.009; SAL–0.5 h vs. MUS–1 h, *p* < 0.001, Figure 1(A4); mPFC: F (2,13) = 7.340, *p* = 0.007, Tukey: SAL–0.5 h vs. MUS–0.5 h, *p* = 0.04, SAL–0.5 h vs. MUS–1 h, *p* = 0.007, Figure 1(B4)).

In NAc, the expression of GluA1 and its phosphorylation were not changed from controls 0.5 h after the EP inhibition, but 1 h later, the expression of GluA1 was decreased when compared with the control group and 0.5 h group (F (2,15) = 8.670, *p* = 0.003, Tukey: SAL–0.5 h vs. MUS–1 h, *p* = 0.005, MUS–0.5 h vs. MUS–1 h, *p* = 0.01, Figure 1(C2)). The phosphorylation of GluA1 at p831 and p845 sites also decreased 1 h after the EP inactivation when compared with 0.5 h after the EP inhibition (p831: VTA: F (2,15) = 6.631, *p* = 0.009, Tukey: MUS–0.5 h vs. MUS–1 h, *p* = 0.006; p845: VTA: F (2,13) = 7.095, *p* = 0.008, Tukey: MUS–0.5 h vs. MUS–1 h, *p* = 0.006, Figure 1(C3,C4)).

In the amygdala, the expression of GluA1 and its phosphorylation at the p831 site were significantly increased 0.5 h and 1 h after the EP inhibition when compared with controls (GluA1: F (2,13) = 12.83, *p* < 0.001, Tukey: SAL–0.5 h vs. MUS–0.5 h, *p* = 0.04, SAL–0.5 h vs. MUS–1 h, *p* < 0.001, Figure 1(D2); p831: F (2,14) = 5.717, *p* = 0.02, Tukey: SAL–0.5 h vs. MUS–0.5 h, *p* = 0.04, SAL–0.5 h vs. MUS–1 h, *p* = 0.02, Figure 1(D3)). The phosphorylation of GluA1 at the p845 site was also increased 1 h after EP inactivation compared to the saline group (F (2,15) = 4.642, *p* = 0.03, Tukey: SAL–0.5h vs. MUS–1 h, *p* = 0.02, Figure 1(D4)).

In LHb, the expression of GluA1 and its phosphorylation at p831 and p845 sites were all decreased 0.5 h after the EP inhibition, but reversed to control levels 1 h after the EP inhibition (GluA1: F (2,15) = 10.75, *p* = 0.001, Tukey: SAL–0.5 h vs. MUS–0.5 h, *p* = 0.01, MUS–0.5 h vs. MUS–1 h, *p* = 0.001, Figure 1(E2); p831: F (2,15) = 14.09, *p* < 0.001, Tukey: SAL–0.5 h vs. MUS–0.5 h, *p* = 0.002, MUS–0.5 h vs. MUS–1 h, *p* < 0.001, Figure 1(E3); p845: F (2,15) = 7.378, *p* = 0.006, Tukey: saline vs. MUS–0.5 h, *p* = 0.02, MUS–0.5 h vs. MUS–1 h, *p* = 0.008, Figure 1(E4)).

These results indicate that alterations in glutamatergic signaling occurs during the onset of SIB, the synaptic transmission in the reward system and mPFC might have decreased, while synaptic transmission in the amygdala might have increased.

### 2.2. Disinhibition of NAc by Picrotoxin Increased SIB Occurrences in Rat

The reciprocal connections between VTA and NAc are associated with reward [35] and motivated behavior [36]. Our previous study found that both activation and inactivation of VTA reduced the incidence of SIB in rats [13]. In this study, we tested whether the manipulation of NAc activity could affect SIB incidence.

**Figure 2:**
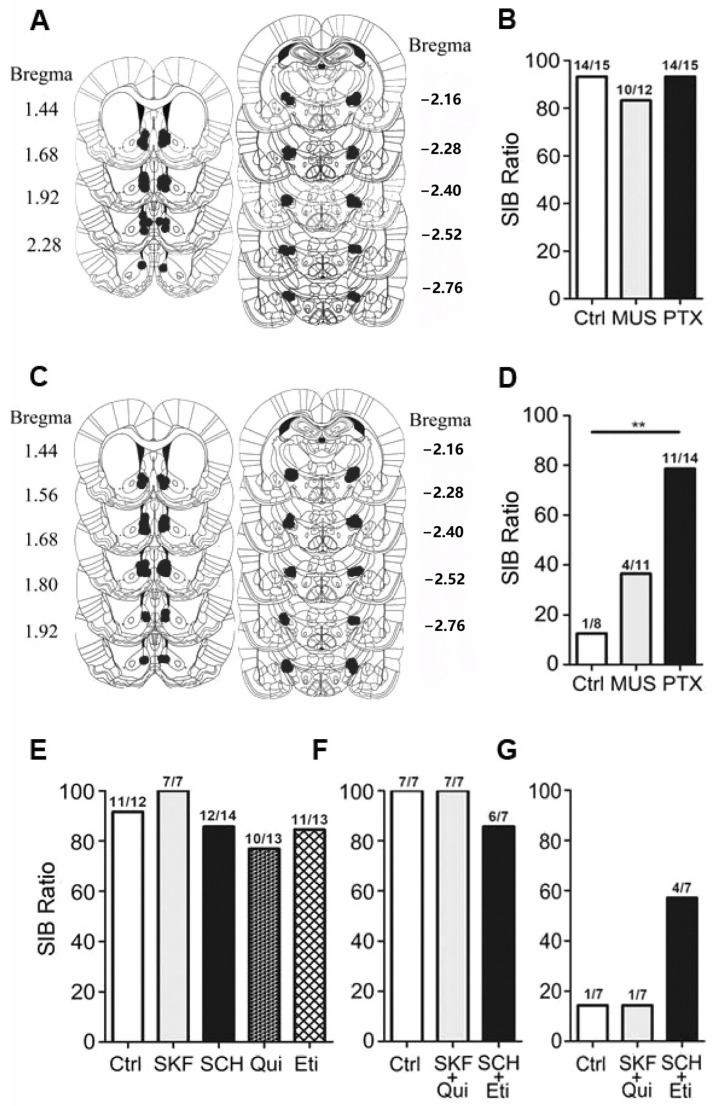
Disinhibition of NAc shell by PTX increased the incidence of SIB. Schematic representation of cannula tracts terminating in the EP (**A**) and NAc shell (**C**). Circle symbols represent the tip of injection tracks. (**B**) Hyperactivation or inactivation of NAc shell did not change the incidence of SIB induced by 1.0 μg muscimol injection in EP. (**D**) Disinhibition of NAc shell increased the incidence of SIB induced by 0.2 μg muscimol injection in EP. (**E**) Dopamine agonist or antagonist manipulation in NAc shell 15 min prior to the 1.0 μg/μL muscimol injection in EP did not change the incidence of SIB. (**F**) Dopamine D1 and D2 agonist or D1 and D2 antagonist manipulation in NAc shell 15 min prior to the 1.0 μg/μL muscimol injection in EP did not change the incidence of SIB. (**G**) Dopamine D1 and D2 agonist or D1 and D2 antagonist manipulation in NAc shell 15 min prior to the 0.2 μg/μL muscimol injection in EP did not change the incidence of SIB. Above each bar is a fraction; the denominator represents the number of rats in that group, and the numerator represents the number of rats in that group that exhibited self-harming behavior. ** *p* < 0.01 for difference between the control and drug-treated groups. NAc: nucleus accumbens; EP: endopeduncular nucleus; MUS: muscimol; PTX: picrotoxin; SCH: SCH-23390; SKF: SKF-38393; Qui: Quinelorane; Eti: Eticlopride; SIB: self-injurious behavior.

First, we investigated the effects of activating or inhibiting the NAc on SIB. A total of 10–15 min before the EP inactivation (muscimol infusion 1.0 μg/μL,1.0 μL/side), 1.0 μL of muscimol (Mus, a GABA_A_ receptor agonist, 1.0 μg), picrotoxin (PTX, a GABA_A_ receptor antagonist, 3.3 μM), or saline was bilaterally delivered to the NAc shell. Self-biting behavior could be found about 0.5–1 h after EP injection and this behavior ended within 6 h in rats with SIB. To our surprise, there was no significant difference in SIB ratio among the three groups (Figure 2B: Ctrl, 14/15; MUS, 10/12; PTX, 14/15. Ctrl vs. MUS: *p* = 0.569; Ctrl vs. PTX: *p* = 1.0, Fisher’s Exact Test).

On the other hand, if SIB was induced with a lower dose of muscimol infusion in the 0.2 EP group (EP infused with 1.0 μL muscimol/hemisphere, 0.2 μg/μL), the SIB incidence was significantly increased in the PTX group compared to the saline group (Figure 2D: Ctrl, 1/8; MUS, 4/11; PTX, 11/14; Ctrl vs PTX: *p* = 0.006, Fisher’s Exact Test), but not the MUS group (Ctrl vs. MUS: *p* = 0.338, Fisher’s Exact Test).

The projection from VTA to NAc mainly consists of dopaminergic fibers, then we examined which kind of dopamine receptors in NAc may contribute to the SIB induced by EP inactivation. In the 1.0 EP group, dopamine D1-like receptor (D1R) and D2-like receptor (D2R) agonist or antagonist SKF-38393 (SKF), SCH-23390 (SCH), Quinelorane (Qui), Eticlopride (Eti) was infused into the NAc shell; the SIB incidence in each group was not significantly different from the saline group (Figure 2E: Ctrl: 11/12; SKF: 7/7; SCH: 12/14; Qui: 10/13; Eti: 11/13; Ctrl vs. SKF: *p* = 1.0; saline vs. SCH: *p* = 1.0; Ctrl vs. Qui: *p* = 0.593; Ctrl vs. Eti: *p* = 1.0, Fisher’s Exact Test). The mixture of D1R and D2R agonist or antagonist also has no effect in the 1.0 EP group (Figure 2F: Ctrl, 7/7; SKF + Qui, 7/7; SCH + Eti, 6/7; Ctrl vs. SKF + Qui: *p* = 1.0; Ctrl vs. SCH + Eti: *p* = 1.0, Fisher’s Exact Test). In the 0.2 EP group, the saline group showed a low SIB incidence; the mixture of dopamine receptor antagonist showed a tendency to increase SIB incidence, which was not statistically different from the saline group (Figure 2G: Ctrl, 1/7; SKF + Qui, 1/7; SCH + Eti, 4/7; saline vs. SKF + Qui: *p* = 1.0; saline vs. SCH + Eti: *p* = 0.266).

Taken together, these results suggested that, when SIB incidence was high, neither modulation of the activity of NAc nor modulation of dopamine receptors in the NAc shell altered SIB ratio in rats. However, when the SIB incidence was low, disinhibition of the NAc shell increased SIB incidence in rats. These results indicate that NAc disinhibition can promote SIB under conditions of lower baseline incidence, while the high-incidence condition is difficult to interpret due to ceiling effects.

### 2.3. Inhibition of the mPFC–NAc Pathway Increased SIB Occurrences in Rat

**Figure 3:**
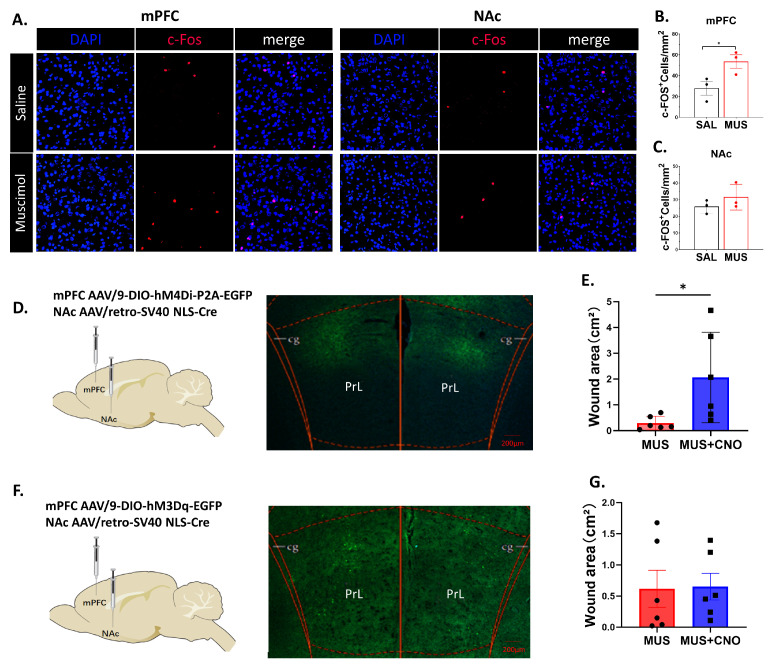
The role of mPFC–NAc pathway in regulating SIB in rats. (**A**) representative images of c-fos expression in the mPFC and NAc during SIB (*n* = 3). (**B**) EP inhibition increased c-fos positive cells in mPFC. The black and red bars represent the control and experimental groups, which received saline and muscimol injections into the EP, respectively. (**C**) EP inhibition had no effects on c-fos expression in NAc. (**D**) Schematic representation of the chemogenetic inhibition strategy. Viral vectors expressing hM4Di were bilaterally injected into the mPFC and NAc to inhibit the mPFC–NAc pathway. The right panel shows a representative fluorescence image of viral expression in the mPFC. PrL: prelimbic cortex, a subregion of the mPFC. (**E**) Chemogenetic inhibition of the mPFC–NAc pathway significantly increased wound severity in rats with SIB, *n* = 6. Red bars indicate SIB induced by muscimol injection into the EP, while blue bars represent the group receiving CNO into the NAc 5 min prior to EP muscimol injection. (**F**) Schematic representation of the chemogenetic activation strategy. Viral vectors expressing hM3Dq were bilaterally injected into the mPFC and NAc to activate the mPFC–NAc pathway. The right panel shows a representative fluorescence image of viral expression in the mPFC. PrL: prelimbic cortex. (**G**) Chemogenetic activation of the mPFC–NAc pathway does not significantly affect SIB in rats, *n* = 6. Red bars indicate SIB induced by muscimol injection into the EP, while blue bars represent the group receiving CNO into the NAc 5 min prior to EP muscimol injection. * *p* < 0.05 for difference between the saline- and CNO-treated groups. mPFC: medial prefrontal cortex; NAc: nucleus accumbens; MUS: muscimol; CNO: Clozapine-N-oxide; SIB: self-injurious behavior.

We next examined c-Fos expression in the mPFC and NAc during SIB. We found that EP inhibition significantly increased the number of c-Fos-positive cells in the mPFC (t (4) = 2.803, *p* = 0.048, Figure 3B). But the c-fos positive cells in NAc was not changed during the SIB. Chemogenetic inhibition of the mPFC–NAc pathway increased wound severity in rats with SIB (*p* = 0.015, Mann–Whitney test, Figure 3E), indicating that mPFC and NAc could be involved in SIB. However, chemogenetic activation of the mPFC–NAc pathway did not change the wound severity in rats with SIB (t (10) = 0.096, *p* = 0.925, Figure 3G).

## 3. Discussion

In this study, we employed a rat SIB model to investigate region-specific changes within the LHb, VTA, NAc, amygdala and mPFC during SIB by measuring the AMPA receptor subunit GluA1 expression and phosphorylation. We found that, after EP inactivation by GABA_A_ receptor agonist muscimol, which induced SIB in rats, expression of GluA1 and phosphorylated GluA1 was reduced in the reward system and mPFC; in contrast, levels were elevated in the amygdala. Modulation of the activities of NAc could change the SIB incidence in rats.

Abnormal synaptic transmission has been widely implicated in various mental disorders, among which SIB exhibits a particularly high prevalence [37,38]. The pathophysiology of SIB is closely linked to neurotransmitter dysregulation and structural abnormalities in neural circuits [39]. Our results showed that, at SIB onset, the total expression and phosphorylation of the core subunits of AMPA receptor GluA1 in the reward (VTA/NAc) and control (mPFC) hubs were depressed, whereas emotion centers (amygdala) exhibited potentiated GluA1 expression and phosphorylation. As normalization of pGluA1 to total GluA1 revealed no significant difference among the groups (Supplementary Figure S1), the data indicate that the proportion of phosphorylated receptors remained unchanged. The apparent increase in pGluA1 is likely attributed to an increase in total protein expression rather than enhanced receptor activation. This suggests that, while the overall expression or synaptic trafficking of GluA1 was modulated, the basal phosphorylation efficiency of the receptor was maintained. Upregulation of total GluA1 expression indicates an increase in the overall number of AMPA receptors on neuronal membranes, typically associated with enhanced excitatory synaptic function. Conversely, downregulation of GluA1 expression suggests reduced AMPA receptor synthesis or accelerated degradation, potentially leading to weakened excitatory synaptic transmission [39,40]. Thus, at the onset of SIB, synaptic weights were redistributed in a region-specific pattern, tilting the network balance from positive to negative valence processing. This is consistent with previous studies, which showed that individuals with NSSI history display heightened reactivity, greater intensity, and prolonged perseveration of negative affect [7], and self-injury could alleviate negative emotions [7].

In our previous study, we have found that the c−fos expression in VTA was significantly increased during the SIB, and modulation of VTA activity by both GABAA receptor agonist and antagonist could partially reduce SIB in rats, indicating an “optimal” activation level of VTA dopaminergic neurons might be required to positively modulate the SIB [14]. VTA and NAc form a loop which plays an essential role in the dopamine reward processing. Thus, it is reasonable to assume that NAc might also be important for SIB occurrence. The NAc shell serves as a critical limbic−motor interface that integrates excitatory inputs from the prefrontal cortex and amygdala, playing a pivotal role in emotional processing and impulse control. As SIB is fundamentally driven by emotional dysregulation and aberrant reward processing, we tested if inhibiting or disinhibiting the NAc shell regulates SIB occurrence in rats. To our surprise, NAc hyperactivation or disinhibition had no effect on the SIB occurrence when EP was inhibited by 1.0 μg/side muscimol (1.0 EP group), which resulted in more than 80% rats showing SIB. Furthermore, neither dopamine D1 nor D2 receptor agonist nor antagonist in NAc could alter the SIB occurrence, and the c-fos expression in NAc was also not changed during the SIB. However, hyperactivation of NAc by picrotoxin increases SIB ratio when EP was inhibited by 0.2 μg/side muscimol (0.2 EP group), at which dose, less than 20% of rats show SIB (Figure 2D); inhibition of NAc by muscimol in the 0.2 EP group had no effects on SIB occurrence. Thus, NAc may exert less regulatory influence on SIB than the VTA. It is possible that VTA neurons projecting to other nuclei may play a role in SIB. This is consistent with studies showing that medications targeting the dopamine system have failed in clinical trials [41]. While dopamine receptors in the NAc shell did not appear to modulate SIB, dopamine neurons in the VTA co-release glutamate; thus, this specific projection may underlie the observed SIB. Therefore, the mechanism underlying the modulating effects of VTA in SIB needs further studies, and why NAc hyperactivation increases SIB ratio when the SIB occurrence was low (0.2 EP group) also needs further studies. The BLA projects densely glutamatergic monosynaptic fibers to the NAc, terminating on the distal dendrites of MSNs, serving as one of the primary pathways for emotion–reward information entering motivational circuits. NAc subpopulations receiving BLA input project to either the VTA or lateral hypothalamus to regulate reward/aversion [42]. Furthermore, the NAc is composed of an NAc shell and NAc core, which have dichotomous efferent and functional roles. Most medium spiny neurons (MSNs) in NAc express either D1-like dopamine receptor or D2-like dopamine receptor only, and these neurons also play different physiologic roles. We may explore the detailed role of NAc neurons in SIB in the future. The mPFC serves as the core hub for integrating self-information and regulating emotions. In this study, c-fos expression in the mPFC was significantly increased during the SIB. This is consistent with fMRI studies showing that, in depressed adolescents with NSSI, activation in the mPFC was relatively increased compared with controls, and those differences are not merely a result of different samples, but most likely associated with the presence or absence of NSSI in adolescents with depression [43]. The mPFC sends extensive projections to the NAc. NAc not only plays roles in reward-seeking, but also plays a critical role in shaping motivated behavior by integrating reward and threat-related signals to guide decision-making and adaptive responses. This pathway has been found to encode both threats and rewards and can dynamically regulate approach and avoidance behaviors in a context-specific manner [44]. We then tested whether activation or inactivation of the mPFC–NAc pathway could modulate SIB ratio in rats. The present study demonstrates that silencing the mPFC–NAc pathway using HM4Di led to a significant increase in SIB in rats. It is possible that weakened inhibitory signals from the mPFC and heightened excitability of the NAc lead to disinhibition of the downstream ventral pallidum/superior spinorostral nucleus circuit, facilitating the release of chewing-aggression motor programs, which need further research. However, it should also be noted that, while the induction of SIB served as a functional validation of the EP targeting based on our previous findings [14], the lack of post hoc histological verification for every subject represents a limitation of this study.

The amygdala is a key limbic region that initiates the threat response; it plays a central role in negative affect. The most common purpose of NSSI is to regulate negative affect [45]. Neuroimaging research has shown that patients with NSSI have heightened amygdala responses to emotional stimuli (both negative and neutral), and that experimental injuries (a painfully cold stimulus or small incision) lead to both attenuation of amygdala responses and to subjective relief of negative affect in adults with NSSI [45]. In this study, the heightened postsynaptic excitability and enhanced synaptic transmission efficiency demonstrated by AMPA receptor phosphorylation in amygdala suggested that amygdala might be quite activated during the SIB, and amygdala could be an important region modulating SIB and it might be a potential target for SIB therapy.

The LHb is a highly conserved region of the vertebrate brain which is implicated in modulating affective disorders such as depression and anxiety; it is also important in modulating avoidance and anti-reward behaviors [46]. In this study, GluA1 and the phosphorylation of GluA1 in LHb were decreased within 0.5 h after EP inhibition but returned to normal 1 h after EP inhibition. In our previous study, we found that modulate the LHb activity could modulate SIB [13], indicating that LHb may play some roles in SIB. The central nucleus of the amygdala (CeA) is the major output for the amygdala complex. A subpopulation of GABAergic neurons expressing somatostatin in the CeA were found to send projections to the LHb to modulate chronic pain and the depressive-like behavior associated with pain [47]. And silencing of the GABAergic CeA–LHb circuit significantly blunted binge-like intake of a 20% ethanol solution in mice [48]. Thus, the CeA–LHb represents a “pain–emotion” regulatory pathway, but its role in SIB requires further investigation.

These findings reveal the fronto-limbic system which processes emotion is critical for the occurrence of SIB. However, this study also has the following limitations: First, the SIB assessment methods are relatively limited. After SIB occurs, the wound area varies significantly among individuals, necessitating the addition of methods such as SIB scoring in the future to optimize the data. Second, drug interventions primarily target the acute phase of the disease and do not provide guidance for disease prevention. Third, although the rat model serves as a preclinical platform for exploring the neurobiological substrates of SIB, and the findings would provide valuable neurobiological insights and therapeutic directions that can be systematically translated to human research and clinical practice, rat SIB is not identical to human NSSI, as the latter is a complex, multifaceted behavior shaped by psychological, social, developmental, and neurobiological factors, with distinct motivational mechanisms (e.g., emotional regulation, interpersonal communication) and clinical presentations. The rat SIB models are primarily designed to recapitulate the core behavioral phenotype of self-harm under controlled experimental conditions, with a focus on neurobiological drivers rather than the full complexity of human NSSI. Furthermore, to minimize potential behavioral variability associated with the estrous cycle in females, which could confound assessments of anxiety and impulse control, this study exclusively utilized male rats. It should be acknowledged, however, that NSSI exhibits significant sex differences in clinical populations. We will incorporate female animals to further elucidate potential sex-specific mechanisms underlying the regulation of this neural circuitry in the future.

## 4. Materials and Methods

### 4.1. Animals

Male adult (8–10 weeks old, about 280 g) Sprague Dawley rats (purchased from the Experimental Animal Institute of Sichuan Academy of Medical Science, Chengdu, China) were randomly assigned to each experimental group. Four rats were housed in one cage (45 cm long, 35 cm wide, and 25 cm height) under a 12 h light/dark cycle. They had food and water available ad lib and were allowed to familiarize with the experimenter and adapt to the laboratory conditions for 1 week before the experiment started. The experiments were carried out during the light phase of the cycle. All experiments were conducted in accordance with the guidelines for the National Care and Use of Animals approved by the National Animal Research Authority of China with protocols approved by the Institutional Animal Care and Use Committees at Kunming University of Science and Technology. Protocols were approved by the Medical Animal Care and Welfare Committee of Kunming University of Science and Technology.

### 4.2. Chemicals Used

Muscimol (Sigma Aldrich, M1523, St. Louis, MO, USA), a GABA_A_ receptor agonist; picrotoxin (Sigma Aldrich, P1675, St. Louis, MO, USA), a GABA_A_ receptor antagonist; SKF-38393 hydrochloride, a D1 receptor agonist; R(+)-SCH-23390 hydrochloride, a D1 receptor antagonist; Quinelorane dihydrochloride, a D2 receptor agonist; and S-(−)-Eticlopride hydrochloride, a D2 receptor antagonist were all procured from Sigma Aldrich (St. Louis, MO, USA).

### 4.3. Establish Rat Model of SIB

First, rats (350–380 g) were anesthetized with pentobarbital sodium salt (60 mg/kg, Merck) and atropine sulfate (0.4 mg/kg), and then placed in a stereotactic apparatus (RWD Life Science, Shenzhen, China). After that, the stainless steel guiding cannula (500 μm outer diameters, 350 μm inner diameters) were positioned 1.0 mm above the bilateral EP (2.5 mm posterior, 3.0 mm lateral, 6.7 mm ventral to bregma) through the holes drilled in the skull; the cannula was then fixed to the skull using dental acrylic. After surgery, rats were housed individually for at least one week of recovery. Following surgery, penicillin was administered intraperitoneally at 200,000 units/kg per dose, every 12 h for a total of two doses, to prevent infection. During all the experiments, we implemented standardized procedures to minimize stress-induced disturbances caused by handling, medication administration, or surgical procedures.

Seven days after the cannula implantation, SIB was induced by bilateral injection of the muscimol into the rat EP. A total of 1.0 μL muscimol solution (1.0 μg/μL or 0.2 μg/μL) was injected bilaterally into EP at a speed of 0.25 μL/min; the cannula was left in place for an additional 3 min to allow diffusion. Control rats received saline injections with the same procedure. To avoid the influence of administration timing and sequence on SIB, drugs were administered alternately. Twenty-four hours after the injection, wound assessments were conducted by an experimenter blinded to the group allocation and treatment conditions. Photographs of each rat were taken to evaluate lesions on various body parts, including the forepaws, chest, abdomen, legs, hind paws, and tail. A standard ruler was placed near the wound and photographed alongside it to serve as a scale bar; the size of the wounds in the photographs was then analyzed using ImageJ (version 2.16.0/1.54p; NIH, Bethesda, MD, USA). First, the wound images were imported; then, using the “Set Scale” function based on the ruler, the wound area was selected (via freehand selections or the wand tool); finally, the wound area was measured, which was used for subsequent statistical analysis. After inspecting the wounds, we disinfected them with povidone-iodine. When a rat’s cumulative wound area exceeded 1 mm^2^, it was classified as SIB-positive. The SIB rate refers to the percentage of rats in the group that exhibit SIB.

### 4.4. Western Blot

Following the bilateral implantation of cannulas into the EP and a 7-day recovery period, rats were divided into saline, MUS–0.5 h, and MUS–1 h groups, *n* = 6 for each group. Subjects received bilateral infusions of saline or muscimol (1 μL). Rats were euthanized and transcardially perfused at 0.5 h after saline administration (SAL–0.5 h), or at 0.5 h (MUS–0.5 h) and 1 h (MUS–1 h) after muscimol injection.

All brain tissue dissections were referenced to bregma as the zero coordinate. Coronal brain sections were cut using a standard rat brain matrix. Dissection boundaries for the mPFC, VTA, LHb, NAc, and amygdala were defined based on anatomical landmarks, including ventricles, characteristic fiber tracts, and distinct nuclear morphology. Surrounding white matter, meninges, blood vessels, and adjacent non-target nuclei were thoroughly removed from all sampled regions to prevent sample contamination.

Brain samples were then lysed in the fresh radio immunoprecipitation assay (RIPA) protein lysis buffer. The protein concentration was determined using Bio-Rad protein assay reagent (Hercules, CA, USA). A total of 10 μL protein lysate containing 10 μg total protein was loaded into each lane. Proteins were separated by sodium dodecyl sulfate–polyacrylamide gel electrophoresis (SDS–PAGE) and transferred onto polyvinylidene fluoride (PVDF) membranes (Millipore Corporation, Billerica, MA, USA). Then, the membranes were soaked in a solution of 3% skim milk (in TBST, pH 7.2, containing 0.1% Tween–20) overnight at 4 °C. The membranes were then incubated by primary antibodies against rabbit anti-GluA1 (Sigma, ab109450, rabbit recombinant mAb [EPR5479], 1:1000), rabbit anti-phospho-AMPA receptor at Ser831/845 antibodies (Sigma, ab109464, rabbit recombinant mAb [EPR1887], ab76321, rabbit recombinant mAb [EPR2148], 1:1000) or β–actin (ab8227, rabbit polyclonal antibody, 1:1000) overnight at 4 °C. After extensive washing, the membranes were incubated with peroxidase conjugated goat anti-mouse or anti-rabbit IgG (Kirkegaard & Perry Laboratories, #474–1806, #474–1506, Gaithersburg, MD, USA). The epitopes were visualized with an enhanced chemiluminescence (ECL) Western blot detection kit (Tanon Corporation, Shanghai, China). Other steps were performed following the instructions of each antibody. Densitometry analysis was performed using ImageJ software. The band intensity of each target protein was normalized to the internal reference β–Actin. Identical electrophoresis, transfer, blocking and antibody incubation conditions were strictly maintained for all independent blots to reduce inter-membrane deviation.

### 4.5. Immunofluorescence

Rats were euthanized by an overdose of 25% ulatane (1 mL/100 g), followed by intra-cardiac perfusion with 200 mL saline and 200 mL 4% paraformaldehyde. Brains were extracted from the skulls and kept in 4% PFA at 4 °C for a week and then kept in 30% sucrose. After dehydration, the brain tissue was sectioned into 20 µm thick slices and mounted onto adhesive slides. The slides were immunostained using the rabbit anti-c–fos (1:500, Cell Signaling, 2250S, Danvers, MA, USA). Immunostains were amplified with appropriate secondary antibodies (1:1000, Invitrogen, A32754, Carlsbad, CA, USA). Fully stained samples were imaged using a Confocal Microscope (Olympus, Spin SR, Hachioji, Japan). Quantitative analysis of c–fos expression in target regions was performed using ImageJ. Manual counting of fluorescent signals was performed to quantify c–fos-positive cells (co-labeled with DAPI). For each brain region of each animal, 3–5 sections were selected, and multiple non-overlapping high-magnification fields of view were chosen from each section for counting. Image acquisition and quantitative analysis were performed by an experimenter who was unaware of the group assignments. Finally, the average number of c–fos-positive cells per unit area was calculated for each brain region across all images from each rat, with a minimum of three image replicates per region.

### 4.6. Histology

Rats were anesthetized and perfused with saline followed by 4% formaldehyde. Subsequently, brains were taken out and fixed in 4% formaldehyde and then 30% sucrose. Brain slices (40 μm thick) were stained with cresyl violet to determine the cannula tips. Animals with wrong cannula position were excluded.

### 4.7. Experimental Design

#### 4.7.1. Experiment 1: Effects of EP Inactivation on Synaptic Modification in VTA, mPFC, NAc, Amygdala and LHb

**Figure 4:**
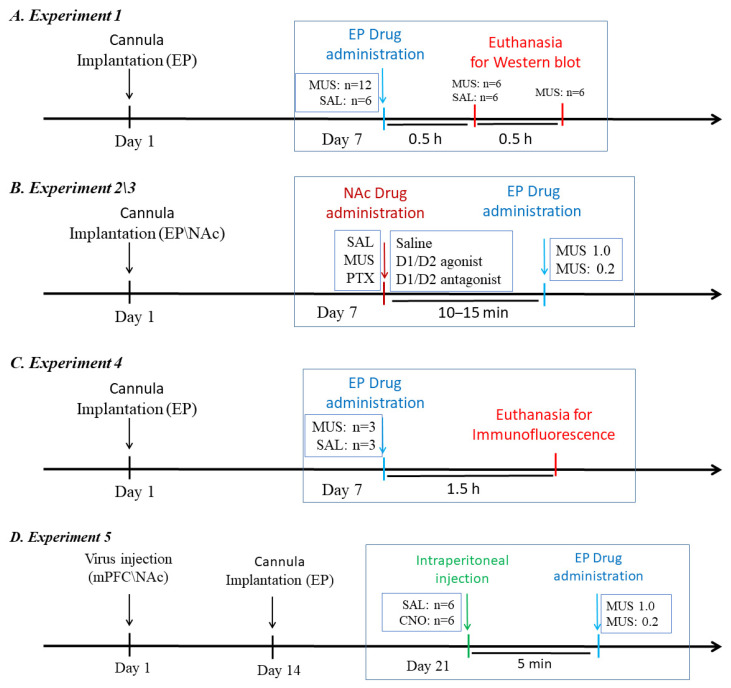
Schedule and design of different experimental protocols. The number of rats used in experiment 1, 4 and 5 are shown on the figures. EP: endopeduncular nucleus; MUS: muscimol; SAL: saline; NAc: nucleus accumbens; mPFC: medial prefrontal cortex; CNO: Clozapine-N-oxide.

As shown in Figure 4A, three groups of rats were used, *n* = 6 for each group. The first group was euthanized 0.5 h after saline injection into EP, the second group was euthanized 0.5 h after the muscimol injection, and the third group was euthanized 1 h after the muscimol injection. The brain areas (VTA, PFC, NAc, amygdala and LHb) were rapidly dissected out, frozen, and stored in a deep freezer at −80 °C until analysis. Tissues were then homogenized and centrifuged in protein lysates for Western blot.

#### 4.7.2. Experiment 2: Effects of NAc Activation or Inactivation on EP Inactivation-Induced SIB

Six groups of rats were used; the number of rats used are as follows (Figure 4B): saline (NAc) + musmol 1.0 μg (EP): *n* = 15; muscimol (NAc) + musmol 1.0 μg (EP): *n* = 12; picrotoxin (NAc) + musmol 1.0 μg (EP): *n* = 15; saline (NAc) + musmol 0.2 μg (EP): *n* = 8; muscimol (NAc) + musmol 0.2 μg (EP): *n* = 11; picrotoxin (NAc) + musmol 0.2 μg (EP): *n* = 14. Rats were anesthetized as described above, then stainless steel guiding cannulas (500 μm outer diameters, 350 μm inner diameters) were positioned 1.0 mm bilaterally above the EP and NAc shell (1.7 mm anterior, 0.8 mm lateral, 6.0 mm ventral to bregma) through the holes drilled in the skull; the cannulas were then fixed to the skull using dental acrylic. One week after surgery, the role of NAc activation or inactivation on SIB was examined. First, 1.0 µL of muscimol (1.0 µg/µL), picrotoxin (3.3 mM) or saline was infused bilaterally into the NAc; 10–15 min later, 1.0 µL of muscimol (1.0 µg/µL or 0.2 µg/µL) was administered bilaterally into the EP to trigger SIB (Figure 2B,D). The doses used are based on our previous study [14]. A total of 24 h after the drug injection, each rat was visually examined and photographed for wounds.

#### 4.7.3. Experiment 3: Effects of Dopamine Receptor Agonist or Antagonist in NAc on EP Inactivation-Induced SIB

Eleven groups of rats were used; the number of rats used are as follows (Figure 4B): saline (NAc) + musmol 1.0 μg (EP): *n* = 12; SKF38393 (NAc) + musmol 1.0 μg (EP): *n* = 7; SCH23390 (NAc) + musmol 1.0 μg (EP): *n* = 14; QUI (NAc) + musmol 1.0 μg (EP): *n* = 13; ETI (NAc) + musmol 1.0 μg (EP): *n* = 13; saline (NAc) + musmol 1 μg (EP): *n* = 7; SKF+QUI (NAc) + musmol 1 μg (EP): *n* = 7; SCH+ETI (NAc) + musmol 1 μg (EP): *n* = 7. saline (NAc) + musmol 0.2 μg (EP): *n* = 7; SKF+QUI (NAc) + musmol 0.2 μg (EP): *n* = 7; SCH+ETI (NAc) + musmol 0.2 μg (EP): *n* = 7. Stainless steel guiding cannulas were positioned above the EP and NAc shell as in experiment 2. After the surgery, rats were housed individually for at least one week of recovery. To examine the role of dopamine in NAc on EP inactivation-induced SIB, 1.0 μL of SKF-38393, SCH-23390, Quinelorane, or Eticlopride was injected individually or with a combination of SKF-38393/Quinelorane and SCH-23390/Eticlopride into the bilateral NAc 10–15 min before the EP inactivation. *n* = 8–15 (Figure 2E–G). A total of 24 h after the drug injection, each rat was visually examined and photographed for wounds.

#### 4.7.4. Experiment 4: Effects of EP Inactivation on c-fos Expression in the mPFC and NAc

A total of 1.5 h after the EP injection with saline (*n* = 3) or muscimol (*n* = 3), rats were euthanized and the brains were extracted from the skulls. After several pretreatments as shown above, the brain tissue was sectioned into 20 µm thick slices for cfos and DAPI staining (Figure 4C).

#### 4.7.5. Experiment 5: Effects of Inactivation or Inhibition the mPFC–NAc Pathway on EP Inactivation-Induced SIB

Rats were bilaterally injected with AAV/9-DIO-hM4D(Gi)-EGFP or AAV/9-DIO-hM3D(Gq)-P2A-EGFP in the mPFC, and rAAV-hSyn-SV40 NLS-Cre Retro in NAc (Figure 4D). After viral injection, the scalp was disinfected and sutured. Fourteen days later, cannulas were implanted bilaterally into the EP. Seven days after the cannula implantation, rats were intraperitoneal injected with saline or Clozapine-N-oxide (CNO, ApexBio, Houston, TX, USA, 34233-69-7, 0.33 mg/mL, i.p. 0.2 mL/20 g); immediately after that, 1.0 μL muscimol (1.0 μg/μL or 0.2 μg/μL) was injected bilaterally into EP to induce SIB. A total of 24 h after the drug injection, each rat was visually examined and photographed for wounds.

### 4.8. Statistical Analyses

All data were expressed as the Mean ± SEM. For GluA1 receptor expression and phosphorylation, One-Way Analysis of Variance (ANOVA) with Tukey’s post hoc tests were used to analyze difference between control group and the groups received EP inactivation 0.5 and 1 h later. Fisher’s Exact Tests were used to compare the incidence of SIB between the saline-treated and drug-treated groups. SIB differences between saline- and CNO-treated groups were analyzed by the two-tailed unpaired *t*-test. When the criteria for normality and homoscedasticity were not met, data were analyzed with nonparametric statistics (Mann–Whitney tests). All data were analyzed using SPSS 13.0. Significance was accepted at *p* < 0.05.

## 5. Conclusions

In conclusion, at the onset of SIB, excitatory neuron transmission in the mPFC and reward related nucleus (VTA and NAc) were decreased, while excitatory neuron transmission in the LHb and amygdala were increased, indicating a neural switch from reward to threat dominance. Although VTA has been found to play an important role in SIB [10], the NAc contributions remain unsettled; NAc activation only promotes SIB when baseline rates are low, and once SIB is frequent, neither NAc silencing nor dopaminergic manipulations are effective, indicating that the VTA–NAc dopamine pathway is necessary but not sufficient for SIB. These findings reveal the critical role of the fronto-limbic system in rats exhibiting SIB, which provide some new insights into the neural mechanism of SIB, and offering a basis for NAc in the treatment of SIB.

## Data Availability

The original contributions presented in this study are included in the article/Supplementary Materials. Further inquiries can be directed to the corresponding author.

## Supplementary Materials

The following supporting information can be downloaded at https://www.mdpi.com/article/10.3390/ijms27146256/s1.

## Abbreviations

The following abbreviations are used in this manuscript.

SIB	self-injurious behavior
NSSI	non-suicidal self-injury
GABA	γ-aminobutyric acid
EP	endopeduncular nucleus
GPi	globus pallidus
LHb	lateral habenula
VTA	ventral tegmental area
mPFC	medial prefrontal cortex
NAc	nucleus accumbens
CNO	Clozapine-N-oxide
MUS	muscimol
PTX	picrotoxin
SCH	SCH-23390
SKF	SKF-38393
D1R	dopamine D1 receptor
D2R	dopamine D2 receptor
Qui	Quinelorane
Eti	Eticlopride
GluA1	glutamate receptor AMPA-type subunit 1
