# Supplementary material for: Nucleus Accumbens Hyperactivity and mPFC–NAc Circuit Dysfunction Promote Self-Injurious Behavior in Rats
Source: Int J Mol Sci. 2026 Jul 14;27(14):6256. doi: 10.3390/ijms27146256 (PMC13410692; doi:10.3390/ijms27146256)
Supplement: Supplementary file 1 [file ijms-27-06256-s001.zip › ijms-4372006-supplementary.pdf]

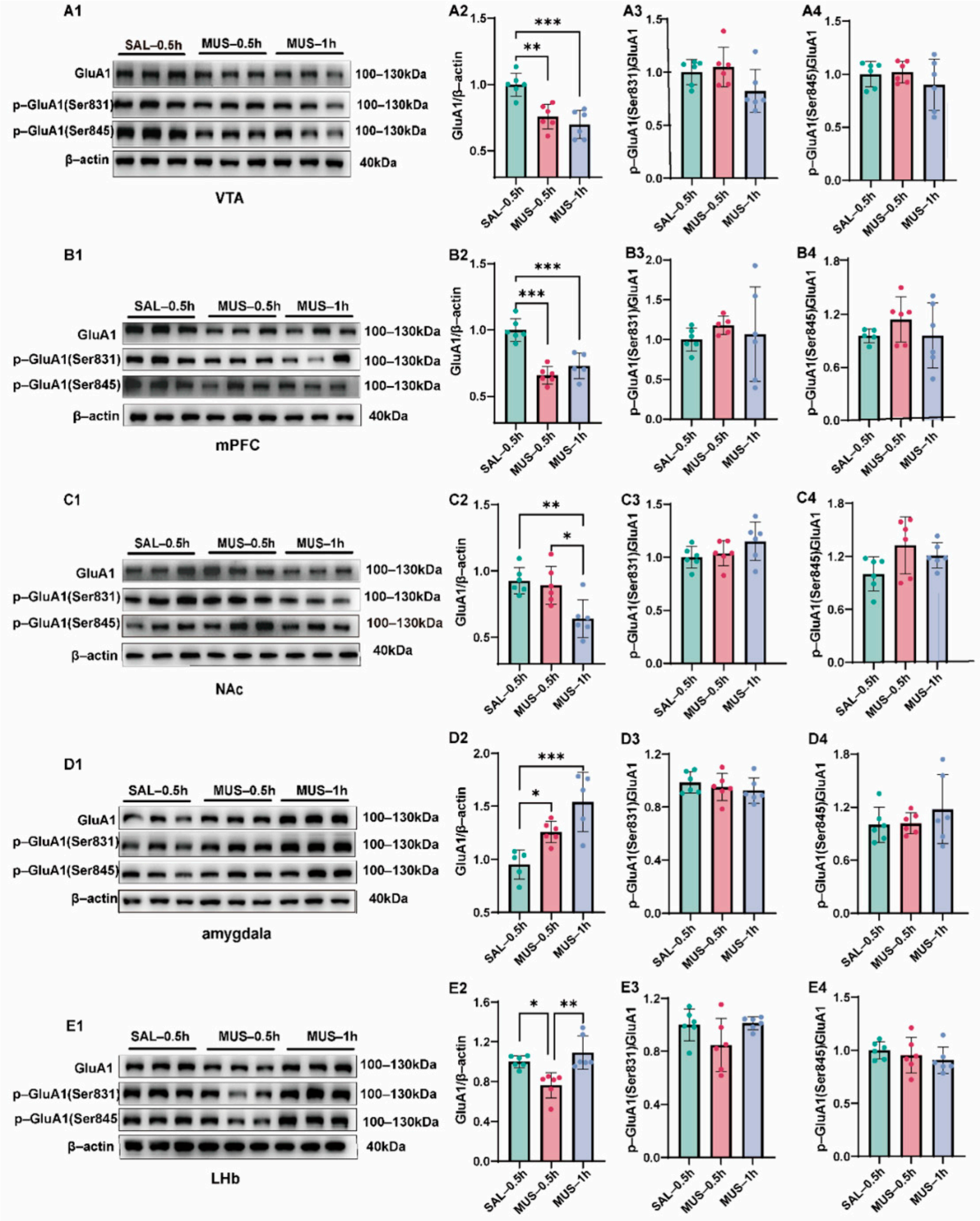

Supplementary Figure S1. Synaptic modifications in VTA, mPFC, NAc, amygdala and LHb at the onset of SIB. The expression of GluA1 and GluA1 phosphorylation relative to total GluA1 in VTA (A1–A4), in mPFC (B1–B4), in NAc (C1–C4), in amygdala (D1–D4), and in LHb were shown (E1-E4). Data were expressed as the mean ± SEM, n=6 rats for each group. *$p<0.05$, **$p<0.01$, ***$p<0.001$ for difference between the control and drug treated group. VTA: ventral tegmental area; PFC: prefrontal cortex; NAc: nucleus accumbens; LHb: lateral habenula; SIB: self-injurious behavior; GluA1: glutamate receptor AMPA type subunit 1.

One and half hours after EP inactivation, the expression of GluA1 significantly decreased in VTA ($F(2,15) = 16.52$, $p<0.001$, Tukey: SAL–0.5 h vs. MUS–0.5 h, $p=0.001$; SAL–0.5 h vs. MUS–1 h, $p<0.001$) and mPFC ($F(2,14) = 28.44$, $p<0.001$, Tukey: SAL–0.5 h vs. MUS–0.5 h, $p=0.001$; SAL–0.5 h vs. MUS–1 h, $p<0.001$). In NAc, the expression of GluA1 was not changed from controls

0.5 h after the EP inhibition, but 1 hour later, the expression of GluA1 was decreased when compared with the control group and 0.5 h group ($F(2,15)=8.670$, $p=0.003$, Tukey: SAL–0.5 h vs. MUS–1 h, $p=0.005$, MUS–0.5 h vs. MUS–1 h, $p=0.01$). In amygdala, the expression of GluA1 were significantly increased 0.5 h and 1 h after the EP inhibition when compared with controls (GluA1: $F(2,13)=12.83$, $p<0.001$, Tukey: SAL–0.5 h vs. MUS–0.5 h, $p=0.04$, SAL–0.5 h vs. MUS–1 h, $p<0.001$). In LHb, the expression of GluA1 was decreased 0.5 h after the EP inhibition, but reversed to control levels 1 h after the EP inhibition (GluA1: $F(2,15)=10.75$, $p=0.001$, Tukey: SAL–0.5 h vs. MUS–0.5 h, $p=0.01$, MUS–0.5 h vs. MUS–1 h, $p=0.001$). However, no significant differences were observed in the ratio of p-GluA1 to total GluA1 across the examined brain regions.
